# Pulsed Field Ablation to Treat Paroxysmal Atrial Fibrillation: Safety and Effectiveness in the AdmIRE Pivotal Trial

**DOI:** 10.1161/CIRCULATIONAHA.124.070333

**Published:** 2024-09-11

**Authors:** Vivek Y. Reddy, Hugh Calkins, Moussa Mansour, Oussama Wazni, Luigi Di Biase, Marwan Bahu, David Newton, Christopher F. Liu, William H. Sauer, Sandeep Goyal, Vivek Iyer, Devi Nair, Charles Athill, Ayman Hussein, Patrick Whalen, Daniel Melby, Andrea Natale

**Affiliations:** 1 Helmsley Electrophysiology Center, Mount Sinai Fuster Heart Hospital, New York, NY (V.Y.R.).; 2 Johns Hopkins Medical Institutions, Baltimore, MD (H.C.).; 3 Massachusetts General Hospital, Boston (M.M.).; 4 Cleveland Clinic Foundation, OH (O.W., A.H.).; 5 Montefiore Health System at Albert Einstein College of Medicine, New York, NY (L.D.B.).; 6 Phoenix Cardiovascular Research Group, AZ (M.B.).; 7 Memorial Health University Medical Center, Savannah, GA (D. Newton).; 8 New York Presbyterian–Weill Cornell Medicine, NY (C.F.L.).; 9 Brigham & Women’s Hospital, Boston, MA (W.H.S.).; 10 Piedmont Heart Institute, Atlanta, GA (S.G.).; 11 Marin Health Medical Center, Larkspur, CA (V.I.).; 12 St. Bernard’s Medical Center & Arrhythmia Research Group, Jonesboro, AR (D. Nair).; 13 San Diego Cardiac Center, CA (C.A.).; 14 Wake Forest Baptist Health, Winston-Salem, NC (P.W.).; 15 Minneapolis Heart Institute, MN (D.M.).; 16 Texas Cardiac Arrhythmia Research Foundation, Austin (A.N.).; 17 Department of Biomedicine and Prevention, Division of Cardiology, University of Tor Vergata, Rome, Italy (A.N.).

**Keywords:** atrial fibrillation, catheters, pulmonary veins

## Abstract

**BACKGROUND:**

Evidence from clinical trials of early pulsed field ablation (PFA) systems in treating atrial fibrillation has demonstrated their promising potential to reduce complications associated with conventional thermal modalities while maintaining efficacy. However, the lack of a fully integrated mapping system, a staple technology of most modern electrophysiology procedures, poses limitations in lesion creation and workflow options. A novel variable-loop PFA catheter integrated with an electroanatomic mapping system has been developed that allows for real-time nonfluoroscopic procedural guidance and lesion indexing as well as feedback of tissue-to-catheter proximity. AdmIRE (Assessment of Safety and Effectiveness in Treatment Management of Atrial Fibrillation With the Bosense-Webster Irreversible Electroporation Ablation System), a multicenter, single-arm, Food and Drug Administration investigational device exemption study, evaluated the long-term safety and effectiveness of this integrated PFA system in a large United States–based drug-refractory symptomatic paroxysmal atrial fibrillation patient population.

**METHODS:**

Using the PFA catheter with a compatible electroanatomic mapping system, patients with drug-refractory symptomatic paroxysmal atrial fibrillation underwent pulmonary vein isolation. The primary safety end point was primary adverse event within 7 days of ablation. The primary effectiveness end point was a composite end point that included 12-month freedom from documented atrial tachyarrhythmia (ie, atrial fibrillation, atrial tachycardia, atrial flutter) episodes, failure to achieve pulmonary vein isolation, use of a nonstudy catheter for pulmonary vein isolation, repeat procedure (except for one redo during blanking), taking a new or previously failed class I or III antiarrhythmic drug at higher dose after blanking, or direct current cardioversion after blanking.

**RESULTS:**

At 30 centers, 277 patients with paroxysmal atrial fibrillation (61.5±10.3 years of age; 64.3% male) in the pivotal cohort underwent PFA. More than 25% of the procedures were performed without fluoroscopy. Median (Q1, Q3) pulmonary vein isolation procedure, fluoroscopy, and transpired PFA application times were 81.0 (61.0, 112.0), 7.1 (0.00, 14.3), and 31.0 (24.8, 40.9) minutes, respectively. The primary adverse event rate was 2.9% (8 of 272), with the most common complication being pericardial tamponade. The 12-month primary effectiveness end point was 74.6%. The 1-year freedom from atrial fibrillation, atrial tachycardia, or atrial flutter recurrence rate after blanking was 75.4%. Substantial improvements in quality of life were observed as early as 3 months after the procedure, concurrent with a reduction in multiple health care use measures.

**CONCLUSIONS:**

AdmIRE confirmed the safety and effectiveness of the variable-loop PFA catheter, with short procedure and PFA application times and low fluoroscopy exposure.

**REGISTRATION:**

URL: https://www.clinicaltrials.gov; Unique identifier: NCT05293639.

**Clinical Perspective**

**What Is New?**

- In AdmIRE (Assessment of Safety and Effectiveness in Treatment Management of Atrial Fibrillation With the BWI IRE Ablation System), researchers evaluated the long-term safety and effectiveness of an integrated pulsed field ablation (PFA) system, consisting of a variable-loop PFA catheter integrated with an electroanatomic mapping system, across 33 US centers.
- In 277 patients with paroxysmal atrial fibrillation (PAF), acute pulmonary vein isolation (PVI) was successful in 100% of cases, first-pass isolation was achieved in 97.5% of targeted veins, and primary effectiveness success was 74.6%.
- Procedure, fluoroscopy, and PFA application times were short, and the primary adverse event rate was low (<3%).

**What Are the Clinical Implications?**

- AdmIRE met the long-term safety and effectiveness end points using the variable-loop PFA catheter, and demonstrated short procedure and PFA application times, as well as low fluoroscopy exposure.

Pulsed field ablation (PFA) is a novel, largely nonthermal cardiac ablation technology that has shown favorable safety and efficacy in single- and multicenter studies evaluating catheter ablation of atrial fibrillation (AF).^1–7^ With a more tissue-selective mechanism of ablation compared with thermal modalities, PFA potentially offers a reduced risk of collateral tissue injury underlying complications such as pulmonary vein (PV) stenosis, atrioesophageal fistula, and permanent phrenic nerve paralysis while maintaining effectiveness.^8–14^

Two PFA catheter systems have completed pivotal trials in the United States, both being multielectrode array catheters fashioned in either a fixed-loop configuration or as an adjustable pentaspline catheter.^7,15^ These PFA catheters met their safety and efficacy end points in their respective pivotal trials, but both technologies have the limitation that they were designed to be primarily guided by x-ray fluoroscopy. In accordance, ad hoc visualization of these PFA catheters on nondedicated electroanatomic mapping (EAM) systems introduces spatial and geometric inaccuracy of the electrode arrays.

A novel PFA catheter system for full high-fidelity integration with a dedicated EAM system has been designed recently. This biphasic bipolar PFA system consists of a multielectrode variable-loop circular catheter (VLCC), used with a multichannel PFA generator. Integration with the mapping system allows for real-time feedback of tissue-to-catheter proximity, which may improve lesion durability.^16^ Preclinical studies have demonstrated good evidence of the tissue preferentiality characteristic of PFA,^14,17,18^ and in a first-in-human European study, the PFA catheter was able to treat patients with paroxysmal AF (PAF) safely and effectively.^19,20^ In accordance, AdmIRE (Assessment of Safety and Effectiveness in Treatment Management of Atrial Fibrillation With the Bosense-Webster Irreversible Electroporation Ablation System; URL: https://www.clinicaltrials.gov; Unique identifier: NCT05293639), a multicenter, single-arm, US Food and Drug Administration (FDA) investigational device exemption pivotal trial, was initiated in the United States to use this PFA VLCC to treat patients with symptomatic drug-resistant PAF. Herein, we report the primary outcomes of AdmIRE, including the safety and effectiveness of this fully integrated PFA system.

## METHODS

### Study Design and Participants

AdmIRE was a multicenter, single-arm, FDA investigational device exemption, prospective clinical evaluation consisting of 2 sequential phases: a short pilot phase for initial safety and effectiveness characterization and a pivotal phase for evaluating safety and effectiveness against prespecified performance goals. Although the pivotal phase included both a roll-in and a main population, this report primarily focuses on the main pivotal patient population, as the pilot phase data have been reported previously.^21^ Enrolled patients underwent first-time pulmonary vein isolation (PVI) and were followed for 12 months after the index ablation procedure.

Eligible patients were between 18 and 75 years of age and had drug-refractory (ie, failed at least one class I or class III antiarrhythmic drug [AAD]) symptomatic PAF. Patients were excluded if diagnosed with persistent AF (>7 days in duration), previous surgical or catheter ablation for AF, or documented left atrial thrombus by imaging within 48 hours of the procedure (a full list of the inclusion and exclusion criteria is presented in Table S1).

The study was approved by the FDA and the ethics committee of each institution, and all patients provided written informed consent. A list of study sites and investigators is provided in Table S2. Johnson & Johnson MedTech has an agreement with the Yale Open Data Access Project to serve as the independent review panel for evaluation of requests for clinical study reports and participant-level data from investigators and physicians for scientific research that will advance medical knowledge and public health. Requests for access to the study data can be submitted through the Yale Open Data Access Project site at http://yoda.yale.edu.

### Ablation Procedure

The ablation system, comprising the multielectrode irrigated VLCC (Varipulse; Biosense Webster, Inc) in combination with the PFA Generator (Trupulse; Biosense Webster, Inc) and the EAM System (CARTO 3; Biosense Webster, Inc), has been described previously (Figure S1).^19,20^ Uninterrupted anticoagulation for at least 3 weeks before ablation was mandatory. Anatomic mapping of the left atrium was performed before ablation using either the VLCC itself or a diagnostic catheter (Pentaray or Lasso; Biosense Webster, Inc); a voltage map was created at physician discretion.

An activated clotting time ≥350 seconds was required before commencing the ablation procedure and was maintained throughout the procedure. For PVI, the number of optimal applications was ≥12 per vein, packaged in salvos of 3 applications per ablation delivered over ≈10 seconds, using all 10 electrodes. Electrodes were deactivated if there was evidence of overlap to prevent electrical arcing. If left atrial arrhythmias were identified during the procedure, ablation outside of the PVs was permitted using the VLCC to deliver segmental applications with ≥6 electrodes or circumferential applications with 10 electrodes. In cases of typical right atrial flutter (AFL), cavotricuspid isthmus (CTI) linear ablation was permitted using a commercially approved radiofrequency ablation catheter. Entrance block was confirmed by elimination of electrograms using a diagnostic multielectrode catheter, upon adenosine/isoproterenol challenge, without a prescribed waiting period. Anticoagulation therapy was required for 2 months after the procedure, and decisions for discontinuation were based on the patient’s stroke risk profile. AAD management during the follow-up period was at the discretion of the investigator.

### Follow-Up

Assessments of arrhythmia recurrence were performed according to protocol using remote rhythm monitoring (KardiaMobile 6L), performed weekly during months 1 to 5, monthly during months 6 to 12, and for any symptomatic events. In addition, 24-hour Holter monitoring was performed at months 6 and 12, and 12-lead ECGs were conducted at months 3, 6, and 12. All event tracings and Holter recordings were independently assessed by a core laboratory (Figure S2).

### Study End Points

#### Primary Safety

The primary safety end point was the occurrence of any of the following primary adverse events (PAEs) within 7 days of the index ablation procedure using the study catheter and PFA generator per protocol: device- or procedure-related death, major vascular access complications or bleeding, myocardial infarction, pericarditis, heart block, permanent phrenic nerve paralysis, stroke, thromboembolism, transient ischemic attack, pulmonary edema, or vagal nerve injury/gastroparesis. PAEs also included cardiac tamponade or perforation occurring up to 30 days after procedure, atrioesophageal fistula occurring up to 90 days after procedure, and PV stenosis occurring anytime during the 12-month follow-up period.

#### Primary Effectiveness

The primary effectiveness end point (PEE) was a composite end point including 12-month freedom from documented (symptomatic or asymptomatic) atrial tachyarrhythmia (AF/atrial tachycardia [AT]/AFL) episodes of ≥30 seconds duration on the basis of rhythm monitoring during the postblanking evaluation period (days 91–365), as well as freedom from other failure modes (ie, failure to achieve entrance block in all PVs; >1 repeat ablation for atrial tachyarrhythmia during the 3-month blanking period or any repeat ablation during the evaluation period; use of a nonstudy catheter to treat the PVs or to ablate left atrial non-PV AF targets during the index procedure or to perform a repeat procedure during the 3-month blanking period; taking new or previously failed class I or III AADs at greater doses during the evaluation period; continuous AF/AT/AFL of unknown origin during the evaluation period; or direct-current cardioversion during the evaluation period for AF/AT/AFL recurrences).

#### Secondary Effectiveness

The secondary end point (ie, qualify of life improvement) was defined as improvement in the Atrial Fibrillation Effect on Quality of Life (AFEQT) total score at 12 months after procedure compared with the baseline score.

#### Additional End Points

Additional study end points included acute procedural success, first-pass isolation (defined as achievement of entrance block evaluated before adenosine/isoproterenol challenge), repeat ablation, freedom from documented AF/AT/AFL recurrence of class I or III AAD, improvement in Canadian Cardiovascular Society–Severity of Atrial Fibrillation score (possible range, class 0 [asymptomatic with respect to AF] to class 4 [AF symptoms have a severe effect on quality of life]) at 12 months after procedure compared with baseline, reduction in cardiovascular hospitalization rates 12 months after procedure compared with 6 months before enrollment, reduction in direct current cardioversion (DCCV) 12 months after procedure compared with 6 months before enrollment, and change in the proportion of patients on AADs at each 3-month follow-up interval compared with baseline.

### Statistical Methods

The trial was designed to compare each co–primary end point against predefined performance goals for patients in the pivotal phase. To calculate the sample size for the primary safety end point, we assumed a true PAE rate of 5% and an attrition rate of 5%. The performance goal was set at 12%, on the basis of a meta-analysis using data from recent trials representing a similar patient population with approved ablation catheters embodying various catheter designs and thermal energy sources. For the PEE, we assumed a true effectiveness failure-free rate of 60% and a 12-month attrition rate of 10%. The performance goal was set at 50% on the basis of the acceptable success rate for PAF ablation in the 2017 HRS/EHRA/ECAS/APHRS/SOLAECE consensus statement.^22^ Based on these assumptions, a sample size of 248 was calculated to be sufficient to provide >80% power for the analysis of each primary safety and effectiveness end point using an exact binomial test at a 1-sided target α of 0.025.

The primary safety end point was analyzed in the modified intent-to-treat population, consisting of all patients in the pivotal cohort who met eligibility and underwent catheter insertion. The primary and secondary effectiveness end points, as well as additional clinical benefits end points, were analyzed in the per-protocol population, consisting of all patients in the pivotal cohort who had no prespecified protocol deviations and underwent the index procedure with the study catheter to treat the study-related arrhythmia. An exact binomial test was performed for the primary safety end point. Event rates were estimated with the use of the Kaplan-Meier method for the PEE and other time-to-event end points. The associated 95% CIs were calculated on the basis of the Greenwood formula. The Fisher exact test was used to determine statistical significance of any heterogeneity for the primary safety and effectiveness end point data by site. The secondary end point (of improvement in quality of life) was evaluated using a paired *t* test. Multiplicity in testing the primary and secondary end points was addressed by using a gatekeeping approach to control the overall type I error at a level of 2.5%. If either of the co–primary end points was not met, then hypothesis testing for the secondary end point would not be conducted.

To evaluate the reduction in health care use and AAD from baseline until the end of the study, repeated measures logistic models were fit to the data for each outcome of interest. Each specified follow-up interval was compared with baseline; multiplicity adjusted *P* values were reported on the basis of the Dunnett test.

Logistic regression modeling was performed to identify potential risk factors associated with recurrence and primary effectiveness failure. Patients with incomplete follow-up or arrhythmia monitoring were considered missing for this analysis. Continuous variables were considered for categorization on the basis of clinically meaningful groups or by their distribution. A multivariable logistic regression model was constructed by incorporating all variables that met the screening criterion of *P*<0.20 in the univariable analysis and retained a *P*<0.05 when modeled jointly. A thorough examination of the correlation matrix between significant variables from the univariable models was conducted to examine potential multicollinearity. If strong collinearity was observed, variables were prioritized on the basis of relevance and interpretability in the model. For each individual significant predictor of PEE or 12-month recurrence, the corresponding PEE or 12-month freedom from recurrence were estimated using the Kaplan-Meier method.

Furthermore, to understand the use of the VLCC in a preablation setting and its effect on procedural measures and effectiveness results, results for patients in whom preablation mapping was performed by the VLCC versus separate diagnostic catheters were summarized descriptively and compared using the Wilcoxon rank sum test for continuous variables and the Fisher exact test for categorical variables.

Statistical analyses were performed using SAS 9.4 or SAS Studio 3.8 (SAS Institute, Inc).

## RESULTS

### Study Participants

**Figure 1.**
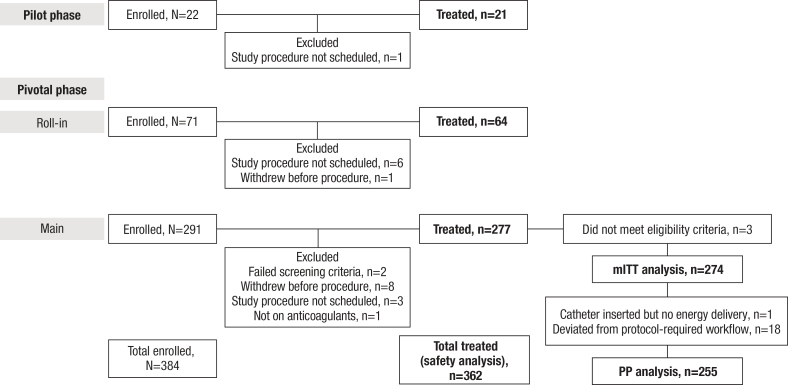
**Patient disposition.** Patient flow chart for both the pilot and pivotal phases. mITT indicates modified intention-to-treat; and PP, per protocol.

Between the pilot and main population pivotal phases, 384 patients with PAF were enrolled across 30 US sites and 39 operators between April and November 2022 (Table S2). Of these, 21 were further treated in the pilot phase, and 277 were treated in the pivotal phase; 5 patients were lost to follow-up. The patient flow is shown in Figure 1. As shown in Table 1, the mean age of the pivotal cohort was 61.5 years, 64.3% were men, and the mean CHA_2_DS_2_-VASC score (congestive heart failure, hypertension, age ≥75 years [doubled], diabetes, stroke [doubled], vascular disease, age 65 to 74 years, and sex category) was 1.7. The mean diagnosis to ablation time was 52.7 months. Baseline characteristics of the pilot cohort were similar (Table S3).

**Table 1.**
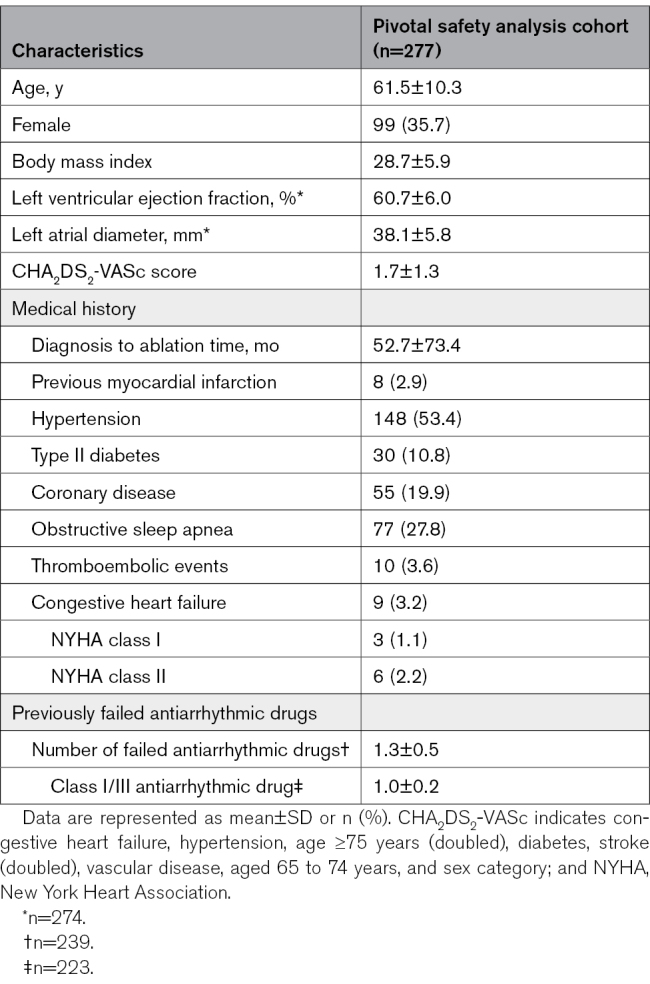
Baseline Characteristics

### Procedural Data

Beyond PVI, the VLCC was used to ablate non-PV sites in 18 patients (6.5%), and a nonstudy catheter was used to ablate non-PV sites in 46 (16.7%; Table S4). The median (Q1–Q3) total procedure times for all procedures and PVI-only procedures were 90.0 (65.0–119.0) and 81.0 (61.0–112.0) minutes, respectively (Table 2). In total, 70.0 (60.0–84.5) PFA applications were delivered per procedure. Among 7 centers, 70 of 277 (25.3%) procedures were performed without fluoroscopy. Procedural characteristics for the pilot cohort were similar to these pivotal cohort metrics (Table S5).

**Table 2.**
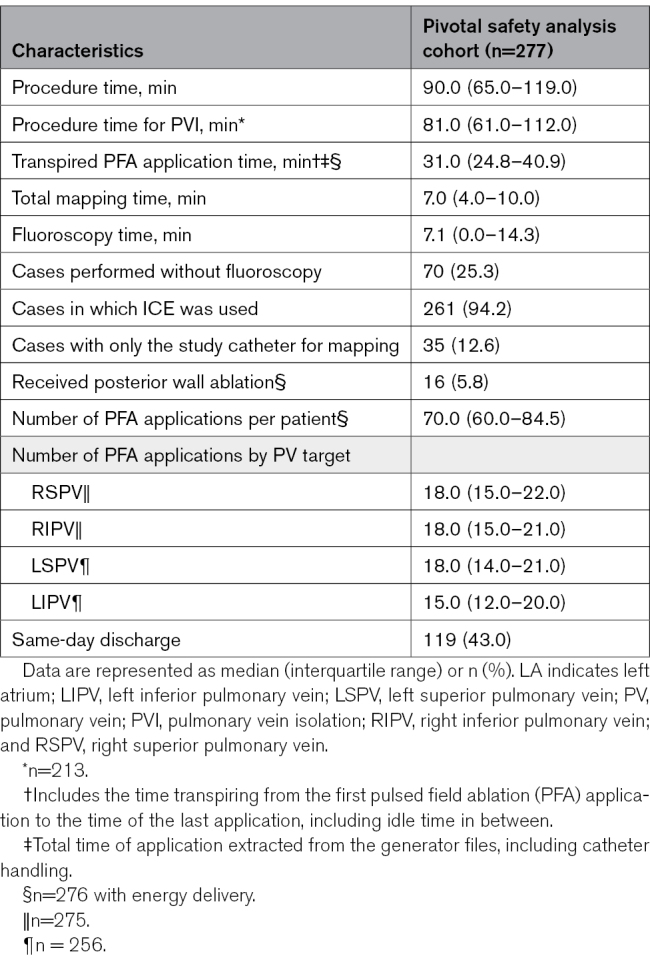
Procedural Characteristics

### Primary Safety End Point

The PAE rate was 2.9% (8 of 272), with an exact one-sided 97.5% upper confidence bound of 5.7%, substantially below the safety performance goal of 12%, indicating primary safety success (Figure 3A). Two patients who had no PAEs before prematurely exiting the study were considered missing outcomes and were excluded from this analysis. No significant heterogeneity in the PAE rate was observed across sites. The PAE rate was similar between those patients treated without fluoroscopy (1 of 69; 1.4% [95% CI, 0–7.8%]) and >0 minutes fluoroscopy (7 of 203; 3.4% [95% CI, 1.4–7.0%]; *P*=0.68).

All 8 PAEs, summarized in Table 3, were procedure-related; among these, 6 (2.2%) were device or pulsed field energy–related, and 5 (1.8%) were pulsed field energy–related. Most PAEs resolved within 3 days of onset, except for one case each of vascular access bleeding (29 days), stroke (32 days), and pericarditis (94 days). There were no reported incidents of device- or procedure-related death, atrioesophageal fistula, coronary spasm, or hemolysis-related renal failure requiring hemodialysis. A summary of serious non-PAEs is provided in Table S6.

**Table 3.**
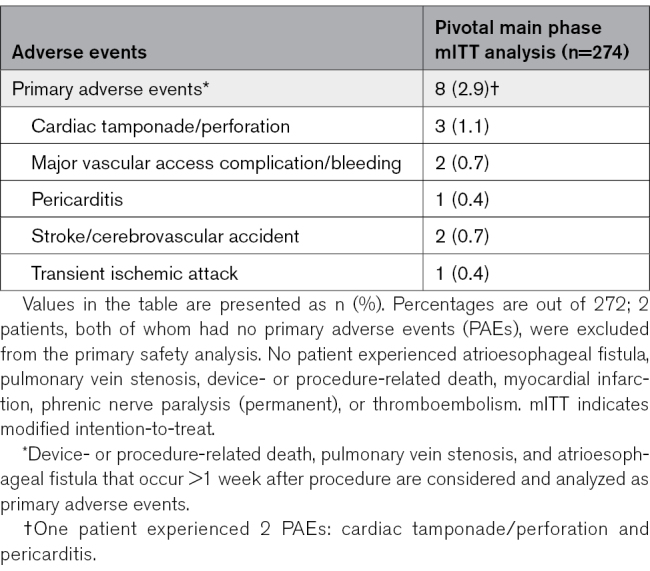
Summary of Primary Adverse Events

Two major vascular access complication or bleeding events occurred, both considered non–device-related. Three cardiac tamponade PAEs occurred, one of which was non–energy-related; these events were successfully treated by either percutaneous pericardiocentesis (n=2) or a surgical window (n=1). Two patients with stroke experienced initial left-sided numbness or migraine, with brain MRIs revealing subacute infarcts: one involving bilateral anterior and posterior circulation territories, and the other the right centrum semiovale and right precentral gyrus. By the 3-month follow-up, the neurological symptoms were largely resolved, although one patient continued a migraine medication. Both patients were receiving uninterrupted anticoagulation 3 weeks before the procedure day, and activated clotting time >350 seconds was maintained before and throughout the procedures.

### Primary Effectiveness End Point

All patients in the pilot and pivotal groups treated with the PFA catheter achieved acute procedural success (100%). First-pass isolation was achieved in 92.5% of patients (236 of 255), encompassing 97.5% of targeted PVs (979 of 1004), with all PVs ultimately isolated using the VLCC catheter alone.

**Figure 2.**
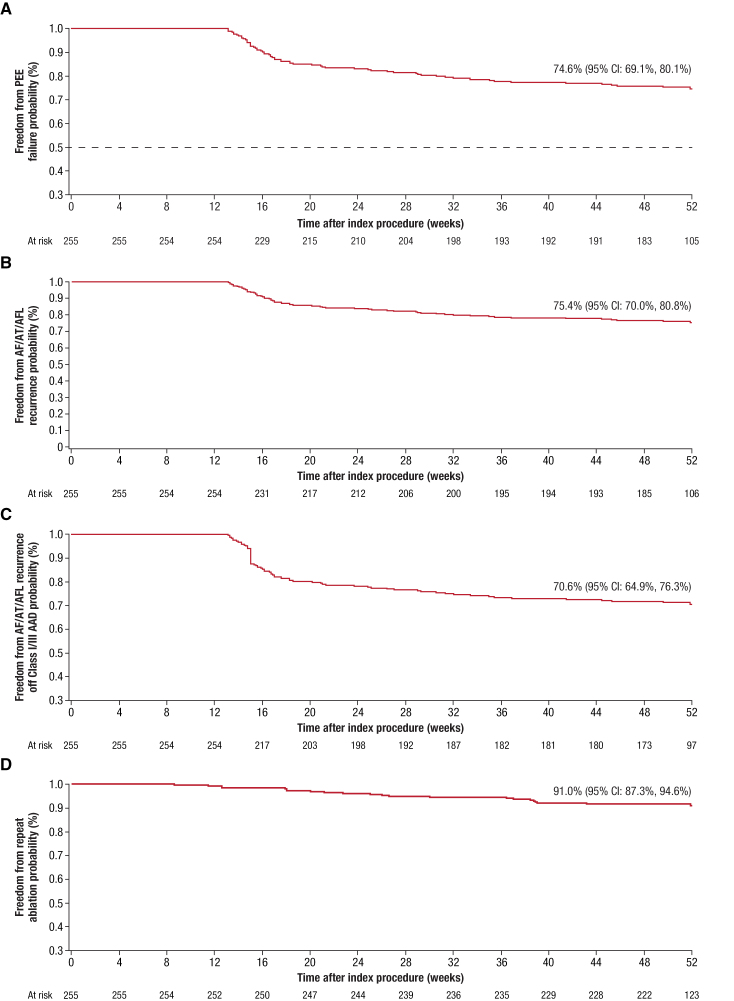
**Kaplan-Meier estimates of effectiveness outcomes.** Kaplan-Meier estimates for 12-month freedom from primary effectiveness end point (PEE) failure (**A**), atrial fibrillation/atrial tachycardia/atrial flutter (AF/AT/AFL) recurrence (**B**), AF/AT/AFL recurrence off class I or III antiarrhythmic drugs (AADs; **C**), and repeat ablation (**D**).

The composite arrhythmia monitoring compliance rate for primary effectiveness ranged from 94.1% to 99.2% at each follow-up visit. The success rate for the composite PEE was 74.6%, with an exact 1-sided 97.5% lower confidence bound of 68.5%, thus exceeding the performance goal of 50%, signifying primary effectiveness success (Figure 2A; Figure S3B). No significant heterogeneity was observed across sites. Primary effectiveness outcomes were similar between those patients treated without fluoroscopy (48 of 66; 72.7% [95% CI, 60.4%–83.0%]) and >0 minutes fluoroscopy (135 of 180; 75.0% [95% CI, 68.0%–81.1%]; *P*=0.74).

The most frequent first-encountered primary effectiveness failure event was postblanking recurrence of AF/AT/AFL, which occurred in 60 patients. The remaining failure events were AAD failure, with use of a new drug in 3 patients.

### Additional Effectiveness Results

The 1-year Kaplan-Meier estimate for freedom from documented AF/AT/AFL recurrence overall and off class I or III AAD was ≥70% (Figure 2B; Figure 2C). Freedom from repeat ablation for atrial tachyarrhythmia was 91.0% (95% CI, 87.3%–94.6%; Figure 2D). Among the 23 patients (9.0%) who underwent a repeat ablation procedure, a total of 91 PVs were targeted in the index procedure; of these, 51 PVs (56.0%) were identified as being electrically reconnected at the repeat ablation procedure.

### Secondary Clinical Benefits and Health Care Use End Points

**Figure 3.**
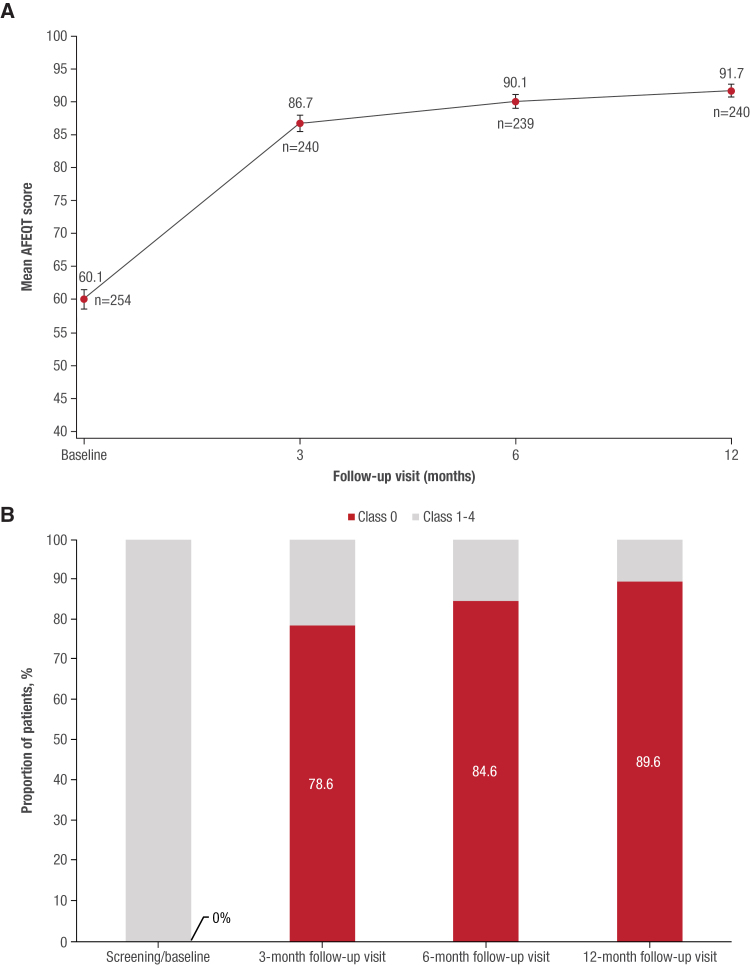
**Quality of life improvement after pulsed field ablation.** Improvements in quality of life after pulsed field ablation with the variable-loop circular catheter as assessed by the mean (±standard error) overall Atrial Fibrillation Effect on Quality of Life (AFEQT) score by follow-up visit (**A**) and mean Canadian Cardiovascular Society–Severity of Atrial Fibrillation score by follow-up visit (**B**; pivotal per-protocol cohort, n=255).

Relative to baseline, there was a significant 32.0-point improvement in the AFEQT score at 12 months (*P*<0.001), with much of this increase observed by the first postablation assessment at 3 months (Figure 3A). The proportion of patients who classified themselves as asymptomatic on the Canadian Cardiovascular Society–Severity of Atrial Fibrillation AF severity scale increased markedly from 0 (0 of 240) at baseline to 89.6% (215 of 240) at 12 months (Figure 3B).

**Figure 4.**
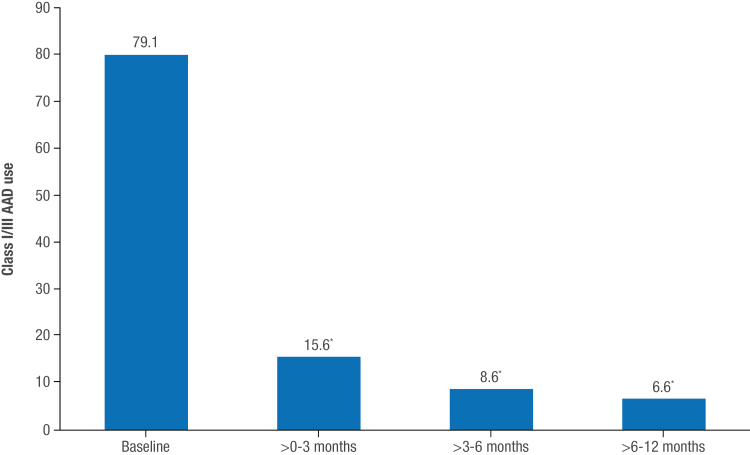
**Use of class I or III antiarrhythmic drugs.** Frequency of antiarrhythmic drug (AAD) use in the pivotal per-protocol cohort (n=255) by follow-up time intervals. ^*^P<0.001 vs baseline.

The rate of DCCV decreased from 4.5% in the 6 months before enrollment to 0.4% during the 6- to 12-month period of follow-up (*P*=0.04), and the rates of cardiovascular hospitalization decreased from 3.3% to 0 over this time period. In addition, compared with the baseline rate (79.1%), class I or III AAD use decreased significantly by 3 months (15.6%; *P*<0.001), with continued decreases observed over 12 months (Figure 4).

### Logistic Regression Analysis for 1-Year Effectiveness

For the composite PEE, a post hoc multivariable logistic regression analysis identified age ≥65 years and diabetes as independent predictors of PEE failure (Figure S4). Also, placing a total number of PFA applications between 73 and 96 for PVI, performed in 85 patients, had lower odds (95% CI) of PEE failure (0.4 [0.2–0.8]) than <73 or >96 PFA applications for PVI. This group demonstrated a freedom from PEE of 85.0%, compared with 74.6% success for the full patient cohort.

### Use of the VLCC for Preablation Mapping

In 12.6% of procedures (35 of 277), the VLCC was used in place of a separate diagnostic catheter for preablation atrial mapping. As expected, this translated to significantly fewer catheter exchanges (*P*<0.001), whereas mapping and procedure times and the 12-month rates of freedom from recurrence were not significantly different (Table 4).

**Table 4.**
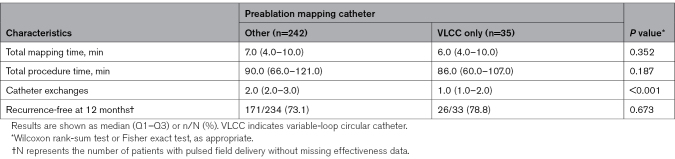
Use of Study Catheter as Preablation Mapping Catheter (Pivotal Safety Analysis Cohort, N=277)

## DISCUSSION

This multicenter FDAinvestigational device exemption pivotal study of the biphasic PFA catheter fully integrated with 3-dimensional mapping in a US cohort demonstrated several important findings: there was an overall favorable safety profile, with a PAE rate of 2.9% across all treated patients; there were no reports of postprocedural thermal esophageal injury, coronary spasm, or hemolysis-related acute kidney injury requiring dialysis; the composite PEE was 75% for all pivotal phase patients; and the mean total procedure time was 80 minutes for PVI, with 25.3% of the procedures performed with no fluoroscopy.

The results from this US pivotal study were consistent with those observed in the earlier INSPIRE (Study for Treatment of Paroxysmal Atrial Fibrillation by Pulsed Field Ablation System with Irreversible Electroporation) of the same PFA system conducted in a limited number of European and Canadian centers. In that study, the 12-month freedom from AF/AT/AFL recurrence was 76% for the full cohort, and 80% when using an optimal number of PFA applications, with no PAEs reported.^19^ The study revealed effectiveness comparable with other recent published clinical trials of PFA, using similar arrhythmia monitoring for PAF, including IMPULSE (A Safety and Feasibility Study of the IOWA Approach Endocardial Ablation System to Treat Atrial Fibrillation), PEFCAT (A Safety and Feasibility Study of the FARAPULSE Endocardial Ablation System to Treat Paroxysmal Atrial Fibrillation) and PEFCAT2 (Expanded Safety and Feasibility Study of the FARAPULSE Endocardial Multi Ablation System to Treat Paroxysmal Atrial Fibrillation) pooled analysis,^3^ the Sphere-9 first-in-human study,^23^ the PULSED AF trial (Pulsed Field Ablation to Irreversibly Electroporate Tissue and Treat AF),^7^ and ADVENT (The FARAPULSE ADVENT Pivotal Trial PFA System vs SOC Ablation for Paroxysmal Atrial Fibrillation),^15^ where 12-month freedom from recurrence ranged between 70% and 79%.

Primary safety events occurred in 2.9% of patients in AdmIRE, consistent with the major safety event rate in the other recent published multicenter clinical trials of PFA, ranging between 0 and 2.5%.^3,7,12,19,24^ It seems likely that some of the traumatic complications of cardiovascular procedures, such as the observed 1.1% rate of pericardial tamponade (including one occurring before pulsed field energy was delivered), will improve considerably as operators move beyond their initial learning curve. Indeed, when an alternate PFA technology, the pentaspline catheter, was first incorporated into the European clinical experience, there was a relatively high rate of tamponade (1.1%) in the initial 1568-patient MANIFEST-PF registry (Multi-National Survey on the Methods, Efficacy, and Safety on the Post-Approval Clinical Use of Pulsed Field Ablation), but this rate dropped dramatically with additional experience to 0.36% in the 17 642-patient MANIFEST-17K cohort.^6,25,26^

In AdmIRE, the incidence of stroke was 0.7%. To this point, it is worth considering that the stroke rate also dropped considerably between the MANIFEST-PF registry and the MANIFEST-17K cohort, largely because of more careful sheath management to avoid inadvertent introduction of air into the systemic circulation.^25,26^ Whereas the pathogenesis of the strokes in AdmIRE is unknown, it is certainly that this stroke rate will improve with operator experience and attention to careful sheath management. It is also possible that additional enhancements to either the catheter or waveform may become necessary. Continued vigilance to potential safety issues is important as future studies are performed.

To our knowledge, AdmIRE is the first pulsed field ablation study to include a substantial subset of procedures performed without the use of any fluoroscopy, which was not driven by protocol; in fact, up to a quarter of study procedures in this trial were performed without fluoroscopic imaging. Exposure to periprocedural radiation is a known health hazard for electrophysiology laboratory staff and patients, elevating the lifetime risk of cognitive impairment and brain malignancy. Wearing a protective shielding apron also contributes to musculoskeletal pain.^27,28^ The European trial using the same VLCC with EAM integration also demonstrated a low mean fluoroscopy time of 8 minutes.^19^ Additional studies using PFA have observed fluoroscopy times ranging from 4 to 26 minutes,^7,15,23^ whereas standard of care procedures have shown 12 to 14 minutes of fluoroscopy time.^5,25,26^ Integration of the VLCC with an EAM system and use of intracardiac echocardiography enabled operators to perform the complete procedure, from transseptal punctures to PVI with PFA, without relying on fluoroscopy; higher rates of no-fluoroscopy procedures with this system might be expected in real-world practice.

Consistent with the rhythm monitoring results, compared with baseline, there were improvements observed in important patient-centric metrics. Regarding quality of life, the 32-point score improvement in the AFEQT score between baseline and 1 year was substantial. For reference, a clinically meaningful increase in the AFEQT score is estimated to be between 6 and 19 points, with the latter representing a conservative estimate, widely accepted as corresponding to a substantial and meaningful improvement in quality of life.^29^ Indeed, this 32-point improvement compares well with the increment in AFEQT scores reported in both the PULSED AF (29.4 points) and ADVENT (30.1 points) trials.^7,15^ In addition, whereas all trial participants were indicated as symptomatic by the Canadian Cardiovascular Society–Severity of Atrial Fibrillation survey before receiving PFA, 90% of these patients viewed themselves as free from AF symptoms by 12 months.

The proportion of patients requiring DCCV or cardiovascular hospitalization was virtually eliminated by the end of the follow-up period. Use of class I or III AADs was reduced from 79.1% at baseline to 6.6% at 12 months, which explains both the low number of drug failures for primary effectiveness and the strong freedom from recurrence off AAD at 70.6%. These end points reflected clinical benefits of PFA experienced by patients in their daily life and reduction in their share of health care use.

Although not mandated by the study protocol, most operators used a dedicated diagnostic catheter for preablation anatomic or voltage mapping. In the subset of cases in which, per operator preference, the VLCC was used for anatomic rendering and mapping, a lower number of catheter exchanges was reported. There was no evidence of compromise in 12-month outcomes. This provides initial feasibility for a potential single-catheter workflow alternative with the VLCC in these patients; this should be explored in future studies.

In ≈6% of procedures, the VLCC was used to create lesions along the left atrial posterior wall. Of note, there were no study failures attributable to the use of a nonstudy catheter in these procedures, nor was there incidence of esophageal injury, suggesting that VLCC might be a versatile regional ablation tool beyond PVI. Indeed, 2 investigator-initiated FDA investigational device exemption trials (VIRTUE [Versatility of a Circular Multielectrode Catheter in the Individualized Recognition & Treatment of Atrial Fibrillation and Related Arrhythmias Using Pulsed Field Energy]; URL: https://www.clinicaltrials.gov; Unique identifier: 06056557; and POLARIS [Safety and Effectiveness of Pulmonary Vein Isolation and Posterior Wall Ablation With Pulsed Field Energy in Patients With Paroxysmal and Persistent AF]; URL: https://www.clinicaltrials.gov; Unique identifier: 06099730) are underway to assess the safety and effectiveness of performing PVI and other ablation in the left atrium with the VLCC in broader patient populations than studied in AdmIRE.^30,31^

### Limitations

AdmIRE used a single-arm study design; direct comparison with other PFA systems or thermal ablation technologies would require additional prospective, randomized controlled studies. AdmIRE included a selected population undergoing an initial ablation procedure for refractory symptomatic PAF and excluded patients with substantial comorbidities that could confound safety and effectiveness outcomes, including persistent AF, uncontrolled heart failure, recent thromboembolism, and substantial pulmonary disease. This selected population may differ from that seen in general clinical practice, where patients are generally older, have a higher CHA_2_DS_2_-VASc score, and have higher rates of heart failure.^5,32–35^ Thus, caution should be exercised when extrapolating these findings to the general population with AF. In addition, the current study contained protocol-driven rhythm monitoring at defined follow-up visits, as well as patient-reported symptomatic recurrences. The absence of continuous monitoring with an implanted device raises the question of accuracy for detecting all episodes of atrial arrhythmia recurrences, which would be required to best assess differences in AF burden before versus after ablation. The primary end point was a composite of events including freedom from atrial arrhythmia recurrences, acute failure, use of a nonstudy catheter, repeat procedures, addition of a class I or III AAD, and cardioversion; however, not all components of this end point are of equal clinical importance. Post hoc analyses on predictors of treatment outcome should be verified in future prospective studies. Results from AdmIRE may not be generalizable to populations outside the United States.

### Conclusions

AdmIRE demonstrated the safety and effectiveness of the variable-loop PFA catheter in a large US pivotal study, with favorable safety and effectiveness results, short procedure and PFA application times, and 3-dimensional mapping integration that facilitated low fluoroscopy exposure.

## ARTICLE INFORMATION

### Acknowledgments

The authors thank the AdmIRE study personnel and patients for their participation and the following individuals for trial execution, statistical analysis, and input during the development of this article: Christina Kaneko, Jaclyn Alcazar, Tara Gomez, Eric Byun, Melissa Mert, Tiffany Tan, Puneet Jatana, Bin Hu, Reecha Sharma, Jesal Parekh, Kelly Boylan, and Kendra McInnis. Michelle Hughes of Lumanity, Inc, provided medical writing and editorial support, funded by Biosense Webster, Inc, under direction of the authors.

### Sources of Funding

This study was funded by Biosense Webster, Inc.

### Disclosures

Dr Reddy reports receiving consulting fees and grant support from Biosense-Webster; unrelated to this article, serves as a consultant for and has equity in Ablacon, Acutus Medical, Affera-Medtronic, Anumana, Apama Medical-Boston Scientific, APN Health, Aquaheart, Atacor, Autonomix, Axon Therapies, Backbeat, BioSig, CardiaCare, Cardiofocus, CardioNXT/AFTx, Circa Scientific, CoRISMA, Corvia Medical, Dinova-Hangzhou DiNovA EP Technology, East End Medical, EPD-Philips, EP Frontiers, Epix Therapeutics-Medtronic, EpiEP, Eximo, Farapulse-Boston Scientific, Field Medical, Focused Therapeutics, HRT, Intershunt, Javelin, Kardium, Keystone Heart, Laminar Medical, LuxMed, Medlumics, Middlepeak, Neutrace, Nuvera-Biosense Webster, Oracle Health, Restore Medical, Sirona Medical, SoundCath, and Valcare; unrelated to this work, has served as a consultant for Abbott, Adagio Medical, Append Medical, AtriAN, BioTel Heart, Biotronik, Boston Scientific, Cairdac, Cardionomic, CoreMap, Fire1, Gore & Associates, Impulse Dynamics, Medtronic, Novartis, Novo Nordisk, Philips, and Pulse Biosciences; and unrelated to this work, has equity in Atraverse, DRS Vascular, Manual Surgical Sciences, Newpace, Nyra Medical, Soundcath, Surecor, and Vizaramed. Dr Calkins receives consulting fees from Biosense Webster and Boston Scientific and payment for honoraria from Boston Scientific and Medtronic. Dr Mansour reports grants from Biosense Webster, Inc, and Boston Scientific; consulting fees from Biosense Webster, Inc, Boston Scientific, Philips, and Medtronic; support for attending meetings/travel from Biosense Webster, Inc, and Boston Scientific; and stock/stock options from NewPace Limited and EPD Solutions. Dr Wazni serves as a consultant for Biosense Webster. Dr Di Biase is a consultant for Biosense Webster, Stereoataxis, and Rhythm Management, and has received speaker honoraria/travel from Biosense Webster, St. Jude Medical (now Abbott), Boston Scientific, Medtronic, Biotronik, Atricure, Baylis, and Zoll. Drs Bahu and Liu have nothing to disclose. Dr Newton serves as a consultant for Biosense Webster. Dr Sauer receives grants from Biosense Webster and Medtronic and consulting fees from Boston Scientific, Biosense Webster, and Abbott. Dr Goyal receives consulting fees from Biosense Webster and Medtronic. Dr Iyer receives consulting fees from Biosense Webster. Dr Nair serves as a consultant for and receives research grants from Abbott, Boston Scientific, Medtronic, Biosense Webster, and Adagio, and receives research grants from Laminar. Dr Athill receives consulting fees from Boston Scientific, Abbott, and Biosense Webster, and payment/honoraria from Zoll and Janssen. Dr Hussein receives research grants from Boston Scientific and serves on steering committees for Biosense Webster. Dr Whalen serves on the advisory board for Biosense Webster. Dr Melby serves as a consultant for and receives research grants from Biosense Webster. Dr Natale serves as a consultant for Abbott, Biosense Webster, Biotronik, Boston Scientific, and iRhythm.

### Supplemental Material

Tables S1–S6

Figures S1–S4

## Supplementary Material

## References

[1] Reddy VY Anic A Koruth J Petru J Funasako M Minami K Breskovic T Sikiric I Dukkipati SR Kawamura I. Pulsed field ablation in patients with persistent atrial fibrillation. J Am Coll Cardiol. 2020;76:1068–1080. doi: 10.1016/j.jacc.2020.07.007 32854842 10.1016/j.jacc.2020.07.007

[2] Reddy VY Anter E Rackauskas G Peichl P Koruth JS Petru J Funasako M Minami K Natale A Jais P. Lattice-tip focal ablation catheter that toggles between radiofrequency and pulsed field energy to treat atrial fibrillation: a first-in-human trial. Circ Arrhythm Electrophysiol. 2020;13:e008718. doi: 10.1161/CIRCEP.120.008718 32383391 10.1161/CIRCEP.120.008718

[3] Reddy VY Dukkipati SR Neuzil P Anic A Petru J Funasako M Cochet H Minami K Breskovic T Sikiric I. Pulsed field ablation of paroxysmal atrial fibrillation: 1-year outcomes of IMPULSE, PEFCAT, and PEFCAT II. JACC Clin Electrophysiol. 2021;7:614–627. doi: 10.1016/j.jacep.2021.02.014 33933412 10.1016/j.jacep.2021.02.014

[4] Reddy VY Neuzil P Koruth JS Petru J Funosako M Cochet H Sediva L Chovanec M Dukkipati SR Jais P. Pulsed field ablation for pulmonary vein isolation in atrial fibrillation. J Am Coll Cardiol. 2019;74:315–326. doi: 10.1016/j.jacc.2019.04.021 31085321 10.1016/j.jacc.2019.04.021

[5] Schmidt B Bordignon S Neven K Reichlin T Blaauw Y Hansen J Adelino R Ouss A Futing A Roten L. European Real-World Outcomes With Pulsed Field Ablation in Patients With Symptomatic Atrial Fibrillation: lessons from the multi-centre EU-PORIA registry. Europace. 2023;25:euad185. doi: 10.1093/europace/euad185 37379528 10.1093/europace/euad185 PMC10320231

[6] Turagam MK Neuzil P Schmidt B Reichlin T Neven K Metzner A Hansen J Blaauw Y Maury P Arentz T. Safety and effectiveness of pulsed field ablation to treat atrial fibrillation: one-year outcomes from the MANIFEST-PF registry. Circulation. 2023;148:35–46. doi: 10.1161/CIRCULATIONAHA.123.064959 37199171 10.1161/CIRCULATIONAHA.123.064959

[7] Verma A Haines DE Boersma LV Sood N Natale A Marchlinski FE Calkins H Sanders P Packer DL Kuck KH; PULSED AF Investigators. Pulsed field ablation for the treatment of atrial fibrillation: PULSED AF pivotal trial. Circulation. 2023;147:1422–1432. doi: 10.1161/CIRCULATIONAHA.123.063988 36877118 10.1161/CIRCULATIONAHA.123.063988 PMC10158608

[8] Cochet H Nakatani Y Sridi-Cheniti S Cheniti G Ramirez FD Nakashima T Eggert C Schneider C Viswanathan R Derval N. Pulsed field ablation selectively spares the oesophagus during pulmonary vein isolation for atrial fibrillation. Europace. 2021;23:1391–1399. doi: 10.1093/europace/euab090 33961027 10.1093/europace/euab090 PMC8427383

[9] Koruth J Kuroki K Iwasawa J Enomoto Y Viswanathan R Brose R Buck ED Speltz M Dukkipati SR Reddy VY. Preclinical evaluation of pulsed field ablation: electrophysiological and histological assessment of thoracic vein isolation. Circ Arrhythm Electrophysiol. 2019;12:e007781. doi: 10.1161/CIRCEP.119.007781 31826647 10.1161/CIRCEP.119.007781 PMC6924932

[10] Koruth JS Kuroki K Kawamura I Brose R Viswanathan R Buck ED Donskoy E Neuzil P Dukkipati SR Reddy VY. Pulsed field ablation versus radiofrequency ablation: esophageal injury in a novel porcine model. Circ Arrhythm Electrophysiol. 2020;13:e008303. doi: 10.1161/CIRCEP.119.008303 31977250 10.1161/CIRCEP.119.008303 PMC7069397

[11] Mansour M Gerstenfeld EP Patel C Natale A Whang W Cuoco FA Mountantonakis SE Gibson DN Harding JD Holland SK. Pulmonary vein narrowing after pulsed field versus thermal ablation. Europace. 2024;26:euae038. doi: 10.1093/europace/euae038 38305503 10.1093/europace/euae038 PMC10875916

[12] Neven K van Es R van Driel V van Wessel H Fidder H Vink A Doevendans P Wittkampf F. Acute and long-term effects of full-power electroporation ablation directly on the porcine esophagus. Circ Arrhythm Electrophysiol. 2017;10:e004672. doi: 10.1161/CIRCEP.116.004672 28487347 10.1161/CIRCEP.116.004672

[13] Reddy VY Koruth J Jais P Petru J Timko F Skalsky I Hebeler R Labrousse L Barandon L Kralovec S. Ablation of atrial fibrillation with pulsed electric fields: an ultra-rapid, tissue-selective modality for cardiac ablation. JACC Clin Electrophysiol. 2018;4:987–995. doi: 10.1016/j.jacep.2018.04.005 30139499 10.1016/j.jacep.2018.04.005

[14] Yavin H Brem E Zilberman I Shapira-Daniels A Datta K Govari A Anic A Wazni O Anter E. A circular multielectrode pulsed-field ablation catheter “Lasso PFA”: lesion characteristics, durability and effect on neighboring structures. Circ Arrhythm Electrophysiol. 2021;14:e009229. 10.1161/circep.120.009229 33417475 10.1161/CIRCEP.120.009229 PMC7909749

[15] Reddy VY Gerstenfeld EP Natale A Whang W Cuoco FA Patel C Mountantonakis SE Gibson DN Harding JD Ellis CR; ADVENT Investigators. Pulsed field or conventional thermal ablation for paroxysmal atrial fibrillation. N Engl J Med. 2023;389:1660–1671. doi: 10.1056/NEJMoa2307291 37634148 10.1056/NEJMoa2307291

[16] Howard B Verma A Tzou WS Mattison L Kos B Miklavcic D Onal B Stewart MT Sigg DC. Effects of electrode-tissue proximity on cardiac lesion formation using pulsed field ablation. Circ Arrhythm Electrophysiol. 2022;15:e011110. doi: 10.1161/CIRCEP.122.011110 36166690 10.1161/CIRCEP.122.011110 PMC9584049

[17] Hsu JC Banker RS Gibson DN Gomez T Berman D Datta K Chen Q Doshi SK. Comprehensive dose-response study of pulsed field ablation using a circular catheter compared with radiofrequency ablation for pulmonary vein isolation: a preclinical study. Heart Rhythm O2. 2023;4:662–667. doi: 10.1016/j.hroo.2023.09.005 37936668 10.1016/j.hroo.2023.09.005 PMC10626186

[18] Hsu JC Gibson D Banker R Doshi SK Gidney B Gomez T Berman D Datta K Govari A Natale A. In vivo porcine characterization of atrial lesion safety and efficacy utilizing a circular pulsed-field ablation catheter including assessment of collateral damage to adjacent tissue in supratherapeutic ablation applications. J Cardiovasc Electrophysiol. 2022;33:1480–1488. doi: 10.1111/jce.15522 35510408 10.1111/jce.15522 PMC9545022

[19] De Potter T Grimaldi M Duytschaever M Anic A Vijgen J Neuzil P Van Herendael H Verma A Skanes A Scherr D; INSPIRE Trial Investigators. Predictors of success for pulmonary vein isolation with pulsed field ablation using a variable loop catheter with 3D mapping integration: complete 12-month outcomes from INSPIRE. Circ Arrhythm Electrophysiol. 2024;17:e012667. doi: 10.1161/CIRCEP.123.012667 38655693 10.1161/CIRCEP.123.012667 PMC11111320

[20] Duytschaever M De Potter T Grimaldi M Anic A Vijgen J Neuzil P Van Herendael H Verma A Skanes A Scherr D; INSPIRE Trial Investigators. Paroxysmal atrial fibrillation ablation using a novel variable-loop biphasic pulsed field ablation catheter integrated with a 3-dimensional mapping system: 1-year outcomes of the multicenter INSPIRE study. Circ Arrhythm Electrophysiol. 2023;16:e011780. doi: 10.1161/CIRCEP.122.011780 36735937 10.1161/CIRCEP.122.011780 PMC10026968

[21] Newton D Natale A Mansour M Nair D Senn T Reddy VY, on behalf of the ADMIRE Trial Investigators. Pulsed field ablation using a variable loop circular catheter with 3D mapping integration: early outcomes of the ADMIRE study. Presented at the AF Symposium, Boston, MA, February 2, 2024.

[22] Calkins H Hindricks G Cappato R Kim YH Saad EB Aguinaga L Akar JG Badhwar V Brugada J Camm J. 2017 HRS/EHRA/ECAS/APHRS/SOLAECE expert consensus statement on catheter and surgical ablation of atrial fibrillation. Europace. 2018;20:e1–e160. doi: 10.1093/europace/eux274 10.1093/europace/eux274 PMC5834122 29016840

[23] Reddy VY Peichl P Anter E Rackauskas G Petru J Funasako M Minami K Koruth JS Natale A Jais P. A focal ablation catheter toggling between radiofrequency and pulsed field energy to treat atrial fibrillation. JACC Clin Electrophysiol. 2023;9:1786–1801. doi: 10.1016/j.jacep.2023.04.002 37227340 10.1016/j.jacep.2023.04.002

[24] Reddy VY Anter E Peichl P Rackauskas G Petru J Funasako M Koruth JS Marinskis G Turagam M Aidietis A. First-in-human clinical series of a novel conformable large-lattice pulsed field ablation catheter for pulmonary vein isolation. Europace. 2024;26:euae090. doi: 10.1093/europace/euae090 38584468 10.1093/europace/euae090 PMC11057205

[25] Ekanem E Reddy VY Schmidt B Reichlin T Neven K Metzner A Hansen J Blaauw Y Maury P Arentz T; MANIFEST-PF Cooperative. Multi-national survey on the methods, efficacy, and safety on the post-approval clinical use of pulsed field ablation (MANIFEST-PF). Europace. 2022;24:1256–1266. doi: 10.1093/europace/euac050 35647644 10.1093/europace/euac050 PMC9435639

[26] Reddy VY Ekanem E. Multi-national survey on the safety of the post-approval use of pulsed field ablation in 17000+ patients (MANIFEST-17K). Presented at the American Heart Association (AHA) Late Breaking & Featured Science Scientific Sessions; Philadelphia, PA; November 11, 2023.

[27] Gaita F Guerra PG Battaglia A Anselmino M. The dream of near-zero x-rays ablation comes true. Eur Heart J. 2016;37:2749–2755. doi: 10.1093/eurheartj/ehw223 27354053 10.1093/eurheartj/ehw223

[28] Orme NM Rihal CS Gulati R Holmes DR Jr Lennon RJ Lewis BR McPhail IR Thielen KR Pislaru SV Sandhu GS. Occupational health hazards of working in the interventional laboratory: a multisite case control study of physicians and allied staff. J Am Coll Cardiol. 2015;65:820–826. doi: 10.1016/j.jacc.2014.11.056 25720626 10.1016/j.jacc.2014.11.056

[29] Dorian P Burk C Mullin CM Bubien R Godejohn D Reynolds MR Lakkireddy DR Wimmer AP Bhandari A Spertus J. Interpreting changes in quality of life in atrial fibrillation: how much change is meaningful? Am Heart J. 2013;166:381–387.e8. doi: 10.1016/j.ahj.2013.04.015 23895823 10.1016/j.ahj.2013.04.015

[30] Massachusetts General Hospital. Safety and Effectiveness of Pulmonary Vein Isolation and Posterior Wall Ablation With Pulsed Field Energy in Patients With Paroxysmal and Persistent AF (POLARIS). ClinicalTrials.gov identifier: NCT06099730. Updated January 16, 2024. https://clinicaltrials.gov/study/NCT06099730

[31] Reddy VY. Versatility of a Circular Multielectrode Catheter in the Individualized Recognition & Treatment of Atrial Fibrillation and Related Arrhythmias Using Pulsed Field Energy (VIRTUE). ClinicalTrials.gov identifier: NCT06056557. Updated October 18, 2023. https://clinicaltrials.gov/study/NCT06056557

[32] Osorio J Miranda-Arboleda AF Velasco A Varley AL Rajendra A Morales GX Hoyos C Matos C Thorne C D’Souza B. Real-world data of radiofrequency catheter ablation in paroxysmal atrial fibrillation: short- and long-term clinical outcomes from the prospective multicenter REAL-AF Registry. Heart Rhythm. 2024;S1547-5271:02524-4. doi: 10.1016/j.hrthm.2024.04.090 10.1016/j.hrthm.2024.04.090 38768839

[33] Lemoine MD Fink T Mencke C Schleberger R My I Obergassel J Bergau L Sciacca V Rottner L Moser J. Pulsed-field ablation-based pulmonary vein isolation: acute safety, efficacy and short-term follow-up in a multi-center real world scenario. Clin Res Cardiol. 2023;112:795–806. doi: 10.1007/s00392-022-02091-2 36131138 10.1007/s00392-022-02091-2 PMC10241704

[34] Metzl MD Hunter TD Thatcher WH Nazari J. Real-world experience comparing zero versus conventional fluoroscopy catheter ablation for the treatment of symptomatic persistent atrial fibrillation. JAFIB-EP. 2023;16:68–74.

[35] Osorio J Hunter TD Rajendra A Zei P Silverstein J Morales G. Predictors of clinical success after paroxysmal atrial fibrillation catheter ablation. J Cardiovasc Electrophysiol. 2021;32:1814–1821. doi: 10.1111/jce.15028 33825242 10.1111/jce.15028

