# Supplementary material for: Pulsed Field Ablation to Treat Paroxysmal Atrial Fibrillation: Safety and Effectiveness in the AdmIRE Pivotal Trial
Source: Circulation. 2024 Sep 11;150(15):1174–86. doi: 10.1161/CIRCULATIONAHA.124.070333 (PMC11458102; doi:10.1161/CIRCULATIONAHA.124.070333)
Supplement: Supplementary file 1 [file cir-150-1174-s001.pdf]

SUPPLEMENTAL MATERIAL

Supplemental Table S1. Study Inclusion and Exclusion Criteria.

Inclusion Criteria

1. Symptomatic paroxysmal AF with:
   a. ≥2 symptomatic AF episodes within last 6 months from enrollment,
   b. ≥1 AF episode electrocardiographically documented within 12 months prior to enrollment. Electrocardiographic documentation may include, but is not limited to ECG, TTM, Holter monitoring, or telemetry strip, and
   c. A physician's note indicating recurrent self-terminating AF within 7 days.
2. Failed ≥1 AAD (Class I or Class III) as evidenced by recurrent symptomatic AF, intolerable side effects to the AAD, or contraindication to the AAD.
3. Age 18-75 years.
4. Willing and capable of providing consent.
5. Able and willing to comply with all pre-, post- and follow-up testing and requirements.

Exclusion Criteria

1. Previously diagnosed with persistent AF (>7 days in duration).
2. AF secondary to electrolyte imbalance, thyroid disease, or reversible or non-cardiac cause (e.g., untreated documented obstructive sleep apnea and acute alcohol toxicity).
3. Previous surgical or catheter ablation for AF.
4. Patients known to require ablation outside the PV ostia and outside the CTI region (e.g., atrioventricular reentrant tachycardia, atrioventricular nodal reentry tachycardia, atrial tachycardia, ventricular tachycardia and Wolff-Parkinson White).
5. Documented severe dilatation of the LA (LAD >50mm) antero-posterior diameter on imaging within 6 months prior to enrollment.
6. Documented LA thrombus by imaging within 48 hours of the procedure.
7. Documented severely compromised LVEF (LVEF <40%) by imaging within 6 months prior to enrollment.
8. Uncontrolled heart failure or NYHA Class III or IV
9. History of blood clotting, bleeding abnormalities or contraindication to anticoagulation (heparin, warfarin, or dabigatran).
10. Documented thromboembolic event (including TIA) within the past 12 months.
11. Previous PCI/MI within the past 2 months.
12. CABG surgery within the past 6 months (180 days).
13. Valvular cardiac surgical/percutaneous procedure (ie, ventriculotomy, atriotomy, valve repair or replacement and presence of a prosthetic valve).
14. Unstable angina within 6 months.
15. Anticipated cardiac transplantation, cardiac surgery, or other major surgery within the next 12 months.

16. Significant pulmonary disease (eg, restrictive pulmonary disease, constrictive or chronic obstructive pulmonary disease) or any other disease or malfunction of the lungs or respiratory system that produces severe chronic symptoms.
17. Significant congenital anomaly or medical problem that in the opinion of the investigator would preclude enrollment in this study.
18. Prior diagnosis of pulmonary vein stenosis.
19. Pre-existing hemi diaphragmatic paralysis.
20. Acute illness, active systemic infection, or sepsis.
21. Presence of intracardiac thrombus, myxoma, tumor, interatrial baffle or patch or other abnormality that precludes catheter introduction or manipulation.
22. Severe mitral regurgitation (regurgitant volume ≥60 mL/beat, regurgitant fraction ≥50%, and/or effective regurgitant orifice area ≥0.40cm^2).
23. Presence of implanted pacemaker or ICD or other implanted metal cardiac device that may interfere with the IRE energy field.
24. Presence of a condition that precludes vascular access (such as IVC filter).
25. Current enrollment in an investigational study evaluating another device or drug.
26. Women who are pregnant (as evidenced by pregnancy test if pre-menopausal), lactating, or who are of child-bearing age and plan on becoming pregnant during the course of the clinical investigation.
27. Life expectancy <12 months.
28. Presenting contra-indications for the devices used in the study, as indicated in the respective IFU.

AF, atrial fibrillation; ECG, electrocardiogram; TTM, transtelephonic monitoring; AAD, antiarrhythmic drug; PV, pulmonary vein; CTI, cavotricuspid isthmus; LA, left atrium; LAD, left atrium diameter; LVEF, left ventricular ejection fraction; NYHA, New York Heart Association; TIA, transient ischemic attack; PCI, percutaneous coronary intervention; MI, myocardial infarction; CABG, coronary artery bypass grafting; ICD, implantable cardioverter-defibrillator; IRE, irreversible electroporation; IVC, inferior vena cava; IFU, instructions for use.

Supplemental Table S2. Study Sites, Principal Investigators, Sub-Investigators, and Enrollment by Site.

Site	Principal Investigators	Sub-Investigators	Enrolled patients	
			Pilot	Pivotal
**Albert Einstein College of Medicine, Bronx, NY**	Luigi Di Biase	Andrew Krummerman		9
**Arrhythmia Research Group, Jonesboro, AR**	Devi Nair	--	3	11
**Baylor Research Institute, Dallas, TX**	Brian DeVille	Mustafa Dohadwala, Rachel Quast, Angela Prosise, Jai Abraham		6
**Brigham & Women's Hospital, Boston, MA**	William Sauer	Paul Zei, Jorge Romero, Thomas Tadros, Sunil Kapur, Bruce Koplan, Usha Tedrow		21
**Cleveland Clinic Foundation, Cleveland, OH**	Ayman Hussein	Oussama Wazni		10
**Emory Saint Joseph's Hospital, Sandy Springs, GA**	Anshul Patel	David De Lurgio		7
**Evanston Community, Evanston, IL**	Mark Metzl	Jose Nazari, Marcela Goldsmith, Grazia Ferroni		5
**Florida Hospital, Orlando, FL**	George Monir	Sambit Mondal		7
**HOAG Memorial Hospital, Newport Beach, CA**	Rajesh S. Banker	Michael Panutich, Heba Al-Shoubaki, Yasmine Lee		6
**Icahn School of Medicine at Mount Sinai, New York City, NY**	Vivek Reddy, William Whang	Abhishek Maan, Srinivas Dukkipati, Daniel Musikantow, Mohit Turagam	4	38
**Johns Hopkins Medical Institutions, Baltimore, MD**	Hugh Calkins	Jonathan Chrispin		15
**Marin General Hospital, Greenbrae, CA**	Vivek Iyer	Sujoya Dey		18
**Massachusetts General Hospital, Boston, MA**	Moussa Mansour	Kevin Heist	3	8

**Medstar Washington Hospital Center, Washington, DC**	Sung W. Lee	David Strouse, Manish Shah		4
**Memorial Health University Medical Center, Savannah, GA**	David Newton	Todd Senn, Abegail Xiang, Jenny Childs	8	23
**Minneapolis Heart Institute, Minneapolis, MN**	Daniel Melby	Jay Sengupta		9
**Morristown Medical Center, Morristown, NJ**	Jonathan S. Sussman	Timothy Mahoney, Michael Katz, Stephen Winters, Jay Curwin, Robert Coyne, Laura Pizzano		8
**New York Presbyterian, New York, NY**	Christopher Liu	Jim Cheung		22
**New York University Langone Medical Center, New York, NY**	Larry Chinitz	Chriag Barbhaiya		2
**Penn Presbyterian Medical Center, Philadelphia, PA**	Benjamin A. D'Souza	—		2
**Phoenix Cardiovascular Research Group, Phoenix, AZ**	Marwan Bahu	Ashish Sadhu		35
**Piedmont Heart Institute, Atlanta, GA**	Sandeep Goyal	Ashish Bhimani, Kimlien Nguyen, Margaret Trupiano, Thomas Deering, Michael Hoosein, Sricharan Chawdhary Kantipudi, Danesh Kella, Ashli Jackson, Sarah Ellison, Kavita Krishnasamy, Bobby Smith, Andrew Wickliffe, Amynah Welji, Ahmadreza Karimianpour,		19
**San Diego Cardiac Center, San Diego, CA**	Charles Athill	Gregory Francisco		12
**Southshore, Bay Shore, NY**	Jason Chinitz	Aamir Sofi		6
**St. Francis Medical Center, Lynwood, CA**	Joseph Levine	David Hoch		2
**Sutter Health, San Francisco, CA**	Christopher Woods	Steven Hao		3

**Texas Cardiac Arrhythmia Research Foundation, Austin, TX**	Andrea Natale	David Burkhardt, Joe Gallinghouse	4	27
**Virginia Commonwealth University, Richmond, VA**	Kenneth Ellenbogen	Jayanthi Koneru, Jordana Kron, Richard Shepard, Pranav Mankad, Gautham Kalahasty		3
**Wake Forest Baptist Health, Winston-Salem, NC**	Patrick Whalen	Prashant Bhave		10
**WakeMed Hospital, Raleigh , NC**	Ashish B. Patel	Kevin Manocha		6

Supplemental Table S3. Baseline Characteristics – Pilot *vs* Pivotal Cohorts.

Characteristics *	Pilot Safety Analysis Cohort (n=21)	Pivotal Safety Analysis Cohort (n=277)
**Age, years**	62.1 ± 10.2	61.5 ± 10.3
**Female**	9 (42.9)	99 (35.7)
**Body mass index**	30.9 ± 10.2	28.7 ± 5.9
**Left ventricular ejection fraction, % †**	61.9 ± 4.9	60.7 ± 6.0
**Left atrial diameter, mm†**	38.6 ± 7.7	38.1 ± 5.8
**CHA_2DS_2-VASc score**	2.0 ± 0.9	1.7 ± 1.3
**Medical history**		
**Diagnosis to ablation time, months**	66.8 ± 115.6	52.7 ± 73.4
**Prior myocardial infarction**	4 (19.0)	8 (2.9)
**Hypertension**	15 (71.4)	148 (53.4)
**Type II diabetes**	3 (14.3)	30 (10.8)
**Coronary disease**	3 (14.3)	55 (19.9)
**Obstructive sleep apnea**	5 (23.8)	77 (27.8)
**Thromboembolic events**	1 (4.8)	10 (3.6)
**Congestive heart failure**	1 (4.8)	9 (3.2)
**NYHA Class I**	0	3 (1.1)
**NYHA Class II**	1 (4.8)	6 (2.2)
**Previously failed antiarrhythmic drug(s)**		
**Number of failed antiarrhythmic drugs ‡**	1.2 ± 0.4	1.3 ± 0.5
**Class I/III antiarrhythmic drug §**	1.0 ± 0.2	1.0 ± 0.2

CHA_2DS_2-VASc, congestive heart failure, hypertension, aged ≥75 years (doubled), diabetes, stroke (doubled), vascular disease, aged 65 to 74 years, and sex category; NYHA, New York Heart Association.

* Data are represented as mean ± standard deviation or n (%).

† Pilot safety analysis cohort, n=21; pivotal safety analysis cohort=274.

‡ Pilot safety analysis cohort, n=21; pivotal safety analysis cohort=276.

§ Pilot safety analysis cohort, n=21; pivotal safety analysis cohort=239.

ǁ n=223. Pilot safety analysis cohort, n=21; pivotal safety analysis cohort=223.

Supplemental Table S4. Ablation Sites Targeted.

Ablation site targeted, n (%)	Pivotal Safety Analysis Cohort (n=276)
**PVs only**	213 (77.2)
**PVs + Non-PV with study catheter** *	18 (6.5)
**Posterior wall – segmental**	16 (5.8)
**Other AF foci**	1 (0.4)
**Other linear lesion**	1 (0.4)
**PVs + non-PV with non-study catheter** *	46 (16.7)
**CTI line**	40 (14.5)
**SVC – linear lesion**	1 (0.4)
**Other AF foci**	3 (1.1)
**Other linear lesions**	3 (1.1)

AF, atrial fibrillation; CTI, cavotricuspid isthmus; PV pulmonary vein; SVC, superior vena cava.

* Patients may contribute to multiple categories if non-PV targets were ablated with different catheters. Patients may have multiple targets ablated with the same catheter.

Table S5. Procedural Characteristics – Pilot *vs* Pivotal Cohorts.

Characteristics *	Pilot Safety Analysis Cohort (n=21)	Pivotal Safety Analysis Cohort (n=277)
**Procedure time, min**	90.0 (76.0-126.0)	90.0 (65.0-119.0)
**Procedure time for PVI only, min** †	91.0 (78.0-139.0)	81.0 (61.0-112.0)
**Transpired PFA application time, min** ‡,§,¶	34.9 (28.2-44.4)	31.0 (24.8-40.9)
**Total mapping time, min**	7.0 (4.0-9.0)	7.0 (4.0-10.0)
**Fluoroscopy time, min**	3.5 (0.0-14.2)	7.1 (0.0-14.3)
**Cases performed without fluoroscopy**	10 (47.6)	70 (25.4)
**Cases with only the study catheter for mapping**	0 (0)	35 (12.6)
**Received posterior wall ablation** ¶	3 (14.3)	16 (5.8)
**Number of PFA applications per patient** ¶	80.0 (60.0-96.0)	70.0 (60.0-84.5)
**Same day discharge**	14 (66.7)	119 (43.0)

LA, left atrium; PFA, pulsed field ablation; PVI, pulmonary vein isolation.

* Numbers in the table are represented as median (IQR) or n (%).

† Pilot safety analysis cohort, n=18; pivotal safety analysis cohort, n=213.

‡ Includes from the time transpiring between the first PFA application to the time of the last application, including idle time in between.

§ Total time of application extracted from the generator files, including catheter handling.

¶ Pilot safety analysis cohort, n=21; pivotal safety analysis cohort, n=276.

Supplemental Table S6. Summary of Serious Non-Primary AEs (Pivotal Cohort, n=277). (A) Early Onset Non-Primary Serious AEs (Occurring 0-7 Days Post-index/Repeat Ablation) and **(B)** Late Onset Non-Primary Serious AEs (Occurring >30 Days Post-index/Repeat Ablation).

A) Early Onset Non-Primary Serious AEs

Relationship to the device/procedure by body system	No. of subjects with events	Event rate, n/N (%)*
Overall	10	10/277 (3.6)
Causal relationship to device	0	0/277 (0)
Probable relationship to device	0	0/277 (0)
Possible relationship to device	0	0/277 (0)
Not device related	10	10/277 (3.6)
Cardiac disorders	3	3/277 (1.1)
Atrial fibrillation	1	1/277 (0.4)
Cardiac arrest	1	1/277 (0.4)
Pericardial effusion	1	1/277 (0.4)
Gastrointestinal disorders	1	1/277 (0.4)
Lower gastrointestinal hemorrhage	1	1/277 (0.4)
Infections and infestations	2	2/277 (0.7)
Diverticulitis	1	1/277 (0.4)
Pneumonia	1	1/277 (0.4)
Injury, poisoning, and procedural complications	1	1/277 (0.4)
Wound secretion	1	1/277 (0.4)
Nervous system disorders	1	1/277 (0.4)
Migraine	1	1/277 (0.4)
Renal and urinary disorders	2	2/277 (0.7)
Urinary retention	2	2/277 (0.7)
Causal relationship to procedure	2	2/277 (0.7)
Cardiac disorders	1	1/277 (0.4)
Cardiac arrest	1	1/277 (0.4)
Renal and urinary disorders	1	1/277 (0.4)
Urinary retention	1	1/277 (0.4)
Probable relationship to procedure	0	0/277 (0)
Possible relationship to procedure	1	1/277 (0.4)
Nervous system disorders	1	1/277 (0.4)
Migraine	1	1/277 (0.4)
Not procedure related	7	7/277 (2.5)
Cardiac disorders	2	2/277 (0.7)
Atrial fibrillation	1	1/277 (0.4)
Pericardial effusion	1	1/277 (0.4)

Gastrointestinal disorders	1	1/277 (0.4)
Lower gastrointestinal hemorrhage	1	1/277 (0.4)
Infections and infestations	2	2/277 (0.7)
Diverticulitis	1	1/277 (0.4)
Pneumonia	1	1/277 (0.4)
Injury, poisoning, and procedural complications	1	1/277 (0.4)
Wound secretion	1	1/277 (0.4)
Renal and urinary disorders	1	1/277 (0.4)
Urinary retention	1	1/277 (0.4)

B) Late Onset Non-Primary Serious AEs

Relationship to the device/procedure by body system	No. of subjects with events	Event rate, n/N (%)*
Overall	16	16/277 (5.8)
Causal relationship to device	0	0/277 (0)
Probable relationship to device	0	0/277 (0)
Possible relationship to device	0	0/277 (0)
Not device related	16	16/277 (5.0)
Blood and lymphatic system disorders	2	2/277 (0.7)
Anemia	2	2/277 (0.7)
Cardiac disorders	2	2/277 (0.7)
Bradycardia	1	1/277 (0.4)
Acute cardiac failure	1	1/277 (0.4)
Endocrine disorders	1	1/277 (0.4)
Thyroid mass	1	1/277 (0.4)
Gastrointestinal disorders	1	1/277 (0.4)
Rectal hemorrhage	1	1/277 (0.4)
General disorders and administration site conditions	2	2/277 (0.7)
Non-cardiac chest pain	1	1/277 (0.4)
Pyrexia	1	1/277 (0.4)
Injury, poisoning, and procedural complications	3	3/277 (1.1)
Ankle fracture	1	1/277 (0.4)
Multiple fractures	1	1/277 (0.4)
Post-procedural hemorrhage	1	1/277 (0.4)
Metabolism and nutrition disorders	1	1/277 (0.4)
Dehydration	1	1/277 (0.4)
Musculoskeletal and connective tissue disorders	1	1/277 (0.4)
Rotator cuff syndrome	1	1/277 (0.4)

Benign, malignant, and unspecified neoplasms	3	3/277 (1.1)
Colon cancer	2	2/277 (0.7)
Lung adenocarcinoma	1	1/277 (0.4)
Nervous system disorders	1	1/277 (0.4)
Presyncope	1	1/277 (0.4)
Respiratory, thoracic, and mediastinal disorders	1	1/277 (0.4)
Acute respiratory failure	1	1/277 (0.4)
Vascular disorders	2	2/277 (0.7)
Aortic aneurysm	1	1/277 (0.4)
Hemorrhage	1	1/277 (0.4)
Peripheral artery aneurysm	1	1/277 (0.4)
Casual relationship to procedure	0	0/277 (0)
Probable relationship to procedure	0	0/277 (0)
Possible relationship to procedure	0	0/277 (0)
Not procedure related	16	16/277 (5.8)
Blood and lymphatic system disorders	2	2/277 (0.7)
Anemia	2	2/277 (0.7)
Cardiac disorders	2	2/277 (0.7)
Bradycardia	1	1/277 (0.4)
Acute cardiac failure	1	1/277 (0.4)
Endocrine disorders	1	1/277 (0.4)
Thyroid mass	1	1/277 (0.4)
Gastrointestinal disorders	1	1/277 (0.4)
Rectal hemorrhage	1	1/277 (0.4)
General disorders and administration site conditions	2	2/277 (0.7)
Non-cardiac chest pain	1	1/277 (0.4)
Pyrexia	1	1/277 (0.4)
Injury, poisoning, and procedural complications	3	3/277 (1.1)
Ankle fracture	1	1/277 (0.4)
Multiple fractures	1	1/277 (0.4)
Post-procedural hemorrhage	1	1/277 (0.4)
Metabolism and nutrition disorders	1	1/277 (0.4)
Dehydration	1	1/277 (0.4)
Musculoskeletal and connective tissue disorders	1	1/277 (0.4)
Rotator cuff syndrome	1	1/277 (0.4)
Benign, malignant, and unspecified neoplasms	3	3/277 (1.1)

**Colon cancer**	2	2/277 (0.7)
**Lung adenocarcinoma**	1	1/277 (0.4)
**Nervous system disorders**	1	1/277 (0.4)
**Presyncope**	1	1/277 (0.4)
**Respiratory, thoracic, and mediastinal disorders**	1	1/277 (0.4)
**Acute respiratory failure**	1	1/277 (0.4)
**Vascular disorders**	2	2/277 (0.7)
**Aortic aneurysm**	1	1/277 (0.4)
**Hemorrhage**	1	1/277 (0.4)
**Peripheral artery aneurysm**	1	1/277 (0.4)

AE, adverse event.

* Event rate is the percentage of patients with the event.

Supplemental Figure S1. PFA System, Catheter, and CARTO Image.

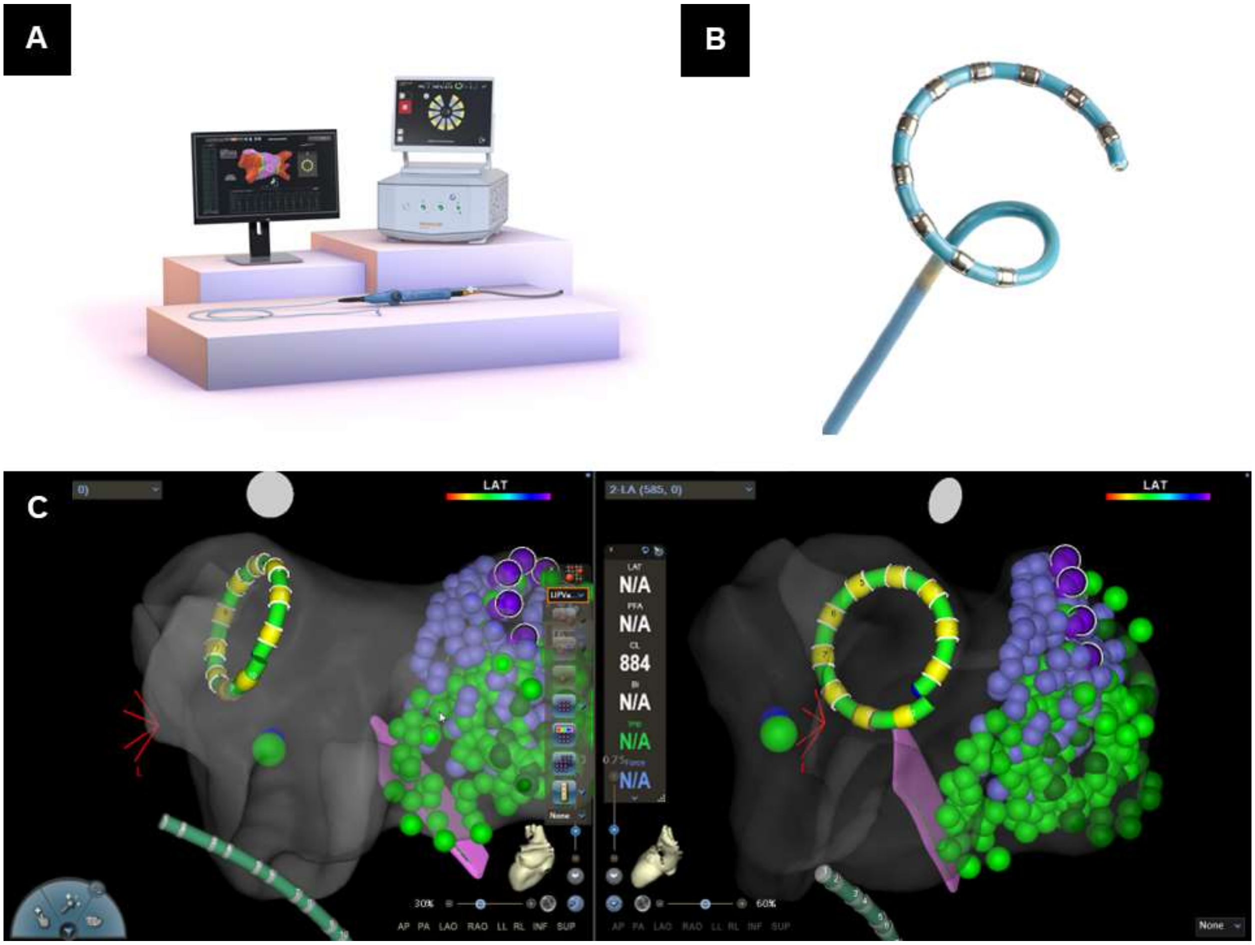

Supplemental Figure S1. PFA System, Catheter, and CARTO Image. (A) PFA System, including the 3D electroanatomical mapping system, PFA generator, and multielectrode variable-loop circular catheter (VLCC). **(B)** Close up of the VLCC. **(C)** CARTO Image from case performed by the integrated PFA System, which reflects the tissue proximity index and electrode overlap detection features. Reprinted from Biosense Webster, Inc. with permission. Copyright ©, Biosense Webster, Inc.

Supplemental Figure S2. Schedule of Assessments.

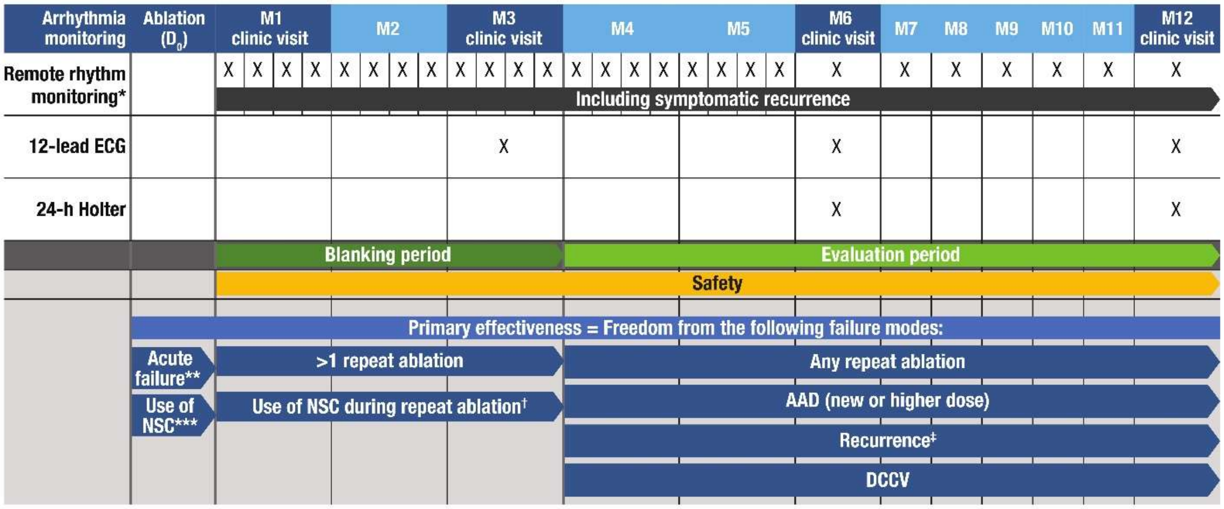

Supplemental Figure S2. Schedule of Assessments. The remote rhythm monitoring (*) includes weekly transtelephonic monitoring (TTM) from months 1–5, monthly TTM from months 6–12, and for symptoms. Acute failure (**) includes failure to confirm entrance block in all pulmonary veins (PVs; except those that are silent and/or cannot be cannulated post-procedure) at end of index procedure. A non-study catheter (NSC; ***) could be used to treat PV targets to achieve isolation of clinically relevant PVs (all PVs except those that are silent and/or cannot be cannulated) and/or to ablate left atrial non-PV atrial fibrillation (AF) targets during the index procedure. During repeat ablation in the blanking period, a non-study catheter (NSC; †) could be used to treat PV targets to achieve isolation of clinically relevant PVs (all PVs except those that are silent and/or cannot be cannulated). Arrhythmia recurrence was defined as continuous AF/AT/AFL of unknown origin by electrocardiographic documentation during the evaluation period. AFL of unknown origin is defined as all AFL except CTI-dependent AFL as confirmed by ECG and entrainment maneuvers in an electrophysiology study. AAD, antiarrhythmic drug; AFL, atrial flutter; AT, atrial tachycardia; CTI, cavotricuspid isthmus; D, day; DCCV, direct current cardioversion; ECG, electrocardiogram; and M, month.

Supplemental Figure S3. Primary Safety and Effectivness Endpoints.

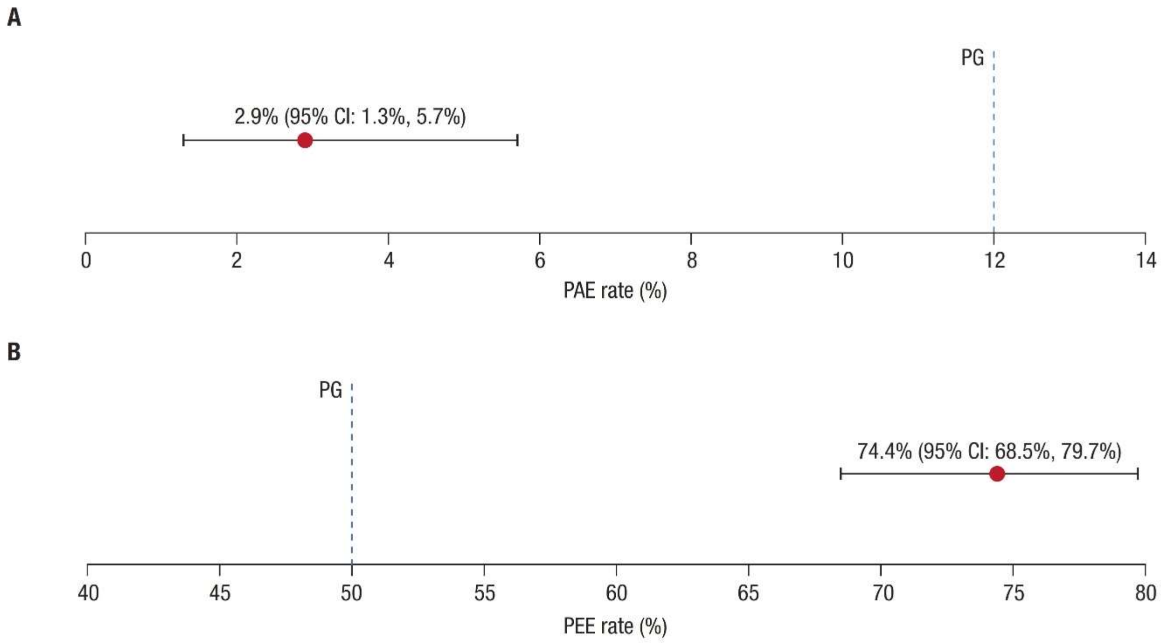

Supplemental Figure S3. Primary Safety and Effectivness Endpoints. Shown are the point estimates, 95% confidence intervals (CI), and performance goals (PG) for the (**A**) primary safety endpoint (pivotal modified intent-to-treat [mITT] analysis set; n = 274) and the (**B**) primary effectiveness endpoint (pivotal per protocol set in patients with non-missing data; n = 246). As depicted, the endpoints were easily met as the upper and lower confidence bounds for the safety and effectiveness endpoints, respectively, were far from the PGs.

Supplemental Figure S4. Predictors of One-Year Effectiveness.

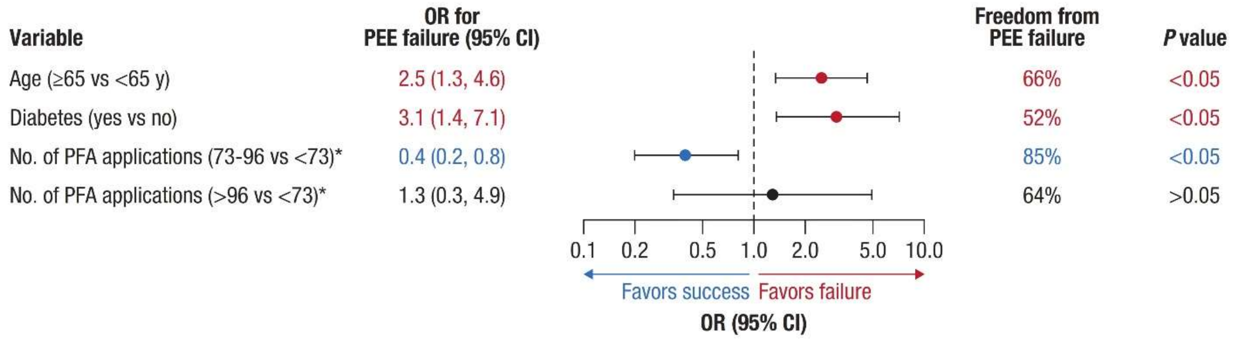

Supplemental Figure S4. Predictors of One-Year Effectiveness. Shown are the predictors of primary effectiveness failure by multivariable analysis in the pivotal per-protocol cohort (n=255). CI, confidence interval; PFA, pulsed field ablation; PVI, pulmonary vein isolation; OR, odds ratio. *Refers to the number of PFA applications for PVI.
